# Ghost Attractors in Spontaneous Brain Activity: Recurrent Excursions Into Functionally-Relevant BOLD Phase-Locking States

**DOI:** 10.3389/fnsys.2020.00020

**Published:** 2020-04-17

**Authors:** Jakub Vohryzek, Gustavo Deco, Bruno Cessac, Morten L. Kringelbach, Joana Cabral

**Affiliations:** ^1^Department of Psychiatry, University of Oxford, Oxford, United Kingdom; ^2^Center for Music in the Brain, Department of Clinical Medicine, Aarhus University, Aarhus, Denmark; ^3^Center for Brain and Cognition, Computational Neuroscience Group, Universitat Pompeu Fabra, Barcelona, Spain; ^4^Department of Neuropsychology, Max Planck Institute for Human Cognitive and Brain Sciences, Leipzig, Germany; ^5^Institució Catalana de Recerca i Estudis Avançats (ICREA), Barcelona, Spain; ^6^Turner Institute for Brain and Mental Health, Monash University, Melbourne, VIC, Australia; ^7^Biovision Team, Université Côte d’Azur, Inria, France; ^8^Life and Health Sciences Research Institute, School of Medicine, University of Minho, Braga, Portugal

**Keywords:** LEiDA, ghost attractors, dynamic functional connectivity, dynamical system theory, functional networks, resting-state

## Abstract

Functionally relevant network patterns form transiently in brain activity during rest, where a given subset of brain areas exhibits temporally synchronized BOLD signals. To adequately assess the biophysical mechanisms governing intrinsic brain activity, a detailed characterization of the dynamical features of functional networks is needed from the experimental side to constrain theoretical models. In this work, we use an open-source fMRI dataset from 100 healthy participants from the Human Connectome Project and analyze whole-brain activity using Leading Eigenvector Dynamics Analysis (LEiDA), which serves to characterize brain activity at each time point by its whole-brain BOLD phase-locking pattern. Clustering these BOLD phase-locking patterns into a set of k states, we demonstrate that the cluster centroids closely overlap with reference functional subsystems. Borrowing tools from dynamical systems theory, we characterize spontaneous brain activity in the form of trajectories within the state space, calculating the Fractional Occupancy and the Dwell Times of each state, as well as the Transition Probabilities between states. Finally, we demonstrate that within-subject reliability is maximized when including the high frequency components of the BOLD signal (>0.1 Hz), indicating the existence of individual fingerprints in dynamical patterns evolving at least as fast as the temporal resolution of acquisition (here *TR* = 0.72 s). Our results reinforce the mechanistic scenario that resting-state networks are the expression of erratic excursions from a baseline synchronous steady state into weakly-stable partially-synchronized states – which we term ghost attractors. To better understand the rules governing the transitions between ghost attractors, we use methods from dynamical systems theory, giving insights into high-order mechanisms underlying brain function.

## Introduction

For healthy human cognition, the brain needs to engage in functionally meaningful activity through an integration of information incoming from various segregated brain areas (Tononi and Edelman, 1998; Sporns et al., 2000). At rest, brain activity has been shown to reveal the spontaneous activation of meaningful functional subsystems, sharing spatial features with networks of brain areas typically activated during task (Beckmann et al., 2005; Fox et al., 2005; Damoiseaux et al., 2006). These spatially activated coalitions of brain regions, dubbed resting-state networks (RSNs), have been remarkably consistent across neuroimaging studies and utilized in describing functional changes in disruptions to the healthy brain functioning (Greicius, 2008; Fox and Greicius, 2010; van den Heuvel and Hulshoff Pol, 2010; Vargas et al., 2013; Kaiser et al., 2015). However, while RSNs represent spatially meaningful information, in order to further investigate the generative mechanisms of RSNs and their functional role, it is important to further characterize their behavior in the temporal domain (Preti et al., 2016; Cabral et al., 2017b).

Indeed, recent advances have focused on how these spatially coherent functional patterns can explain the complex brain dynamics evolving in time (Chang and Glover, 2010; Hutchison et al., 2013; Allen et al., 2014). However, the most appropriate way to characterize network dynamics at the whole brain level is still unclear. The most common approach to dynamic functional connectivity (dFC) has been the sliding-window method, which describes statistical relationship between brain regions in successive intervals of time and generates recurrent states of functional connectivity using unsupervised learning (Hutchison et al., 2013; Allen et al., 2014; Calhoun et al., 2014). However, the choice of the “window” size introduces limitations which hinders the temporal resolution as well as statistical validation (Hindriks et al., 2016; Preti et al., 2016). To overcome these caveats, recent development has focused on describing single frame functional connectivity [FC(t)] either by considering BOLD co-activations (Karahanoğlu and Van De Ville, 2015; Tagliazucchi et al., 2016) or BOLD phase coherence (Glerean et al., 2012; Cabral et al., 2017b). Framewise co-activation analysis considers the brain regions with BOLD signal above a certain threshold before clustering into distinct FC patterns (Karahanoğlu and Van De Ville, 2015; Tagliazucchi et al., 2016). While it allows for higher temporal resolution, it is still dependent on the choice of the threshold as well as limited to describing simultaneous (in-phase) activations. On the other hand, phase coherence techniques represent the time instances as relative phase relationships between brain regions and thus do not require thresholding and are sensitive to phase-shifted patterns (Glerean et al., 2012; Cabral et al., 2017b).

To overcome issues with high data dimensionality, Cabral and colleagues have proposed to represent the instantaneous relationships between brain regions using the largest magnitude eigenvector of BOLD phases (a *1xN* vector for each time point) instead of the *NxN* phase synchronization matrix (Cabral et al., 2017b). Notably, Leading Eigenvector Dynamic Analysis (LEiDA) has been shown not only to improve clustering performance, but to consistently capture meaningful BOLD phase-locking states (PL-states) that closely overlap with previously-described functional subsystems (Cabral et al., 2017b; Figueroa et al., 2019; Lord et al., 2019). By representing whole-brain activity over time as a succession of discrete PL states, it is possible to quantify the fractional occupancy, the probability of transition as well as the Dwell Time of individual states. Importantly, these measures have shown to be significantly related with cognitive performance (Cabral et al., 2017b), to be altered in clinical populations of patients suffering with major depressive disorder (Figueroa et al., 2019), as well as to describe the network-specific modulation of resting-state activity by the psychoactive compound psilocybin (Lord et al., 2019). As such, LEiDA opens up as a useful tool to quantitatively characterize individual fingerprints in dynamic functional connectivity, reinforcing a mechanistic scenario proposed by theoretical works where RSNs are the expression of a repertoire of BOLD FC configurations emerging from complex non-linear interactions in the whole-brain network (Ghosh et al., 2008; Cabral et al., 2011; Deco and Jirsa, 2012; Deco et al., 2013; Haimovici et al., 2013; Hansen et al., 2015).

Here, we explore this mechanistic hypothesis using the mathematical formalism from dynamical systems theory and Markov chains in order to characterize the spatio-temporal dynamics of spontaneous brain activity in terms of probabilistic trajectories between recurrent BOLD phase-locking patterns. We validate the functional role of the patterns obtained by comparing them with known RSNs. Furthermore, we evaluate the stability of BOLD phase locking states based on their Fractional Occupancy, Dwell Times and Transition probabilities. While previous works have applied LEiDA to condition-specific datasets, with reduced sample sizes, here we make secondary use of a large open source dataset of healthy participants, demonstrating the reliability of the yielded metrics across subjects and consecutive fMRI recording sessions.

## Materials and Methods

### Data

All data used in this work comes from a publicly available database – the Human Connectome Project, WU-Minn Consortium (Principal Investigators: David Van Essene and Kamil Ugurbil; 1U54MH091657) with funding from the sixteen NIH Institutes and Centers supporting the NIH Blueprint for Neuroscience Research; and by the McDonell Center for Systems Neuroscience at Washington University.

#### Participants

100 unrelated subjects [mean age 29.5 years old, 55% females (Glasser et al., 2013)].

#### Neuroimaging HCP Acquisition

Each participant underwent four resting-state fMRI sessions lasting 14 min 30 s with a repetition time (TR) of 0.72 s, on a 3-T connectome Skyra scanner (Siemens) – two during the first day and two during the second day. The 2 fMRI sessions acquired on the same day differ only in the oblique axial acquisition phase encoding, one being from Left to Right (LR) and the other from Right to Left (RL). The acquisition and pre-processing of the data is fully described in detail at the HCP website https://www.humanconnectome.org/. Here, we used the fMRI data acquired on the first day of scanning. One subject was excluded because one session was missing. In total, two same-day resting-state fMRI sessions from 99 of the 100 unrelated subjects’ sessions were used for the analysis.

#### Parcellation

**FIGURE 1:**
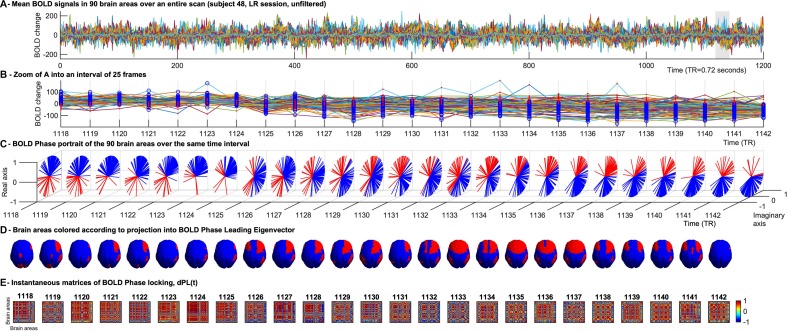
Time-evolving patterns of BOLD Phase Dynamics. **(A)** BOLD signals from a representative fMRI scan of the HCP dataset averaged over all voxels within each region of interest (ROI). ROIs were defined using an anatomically-based parcellation scheme (AAL) covering the entire brain (here excluding the cerebellum). **(B)** To illustrate BOLD phase dynamics, we select a representative interval of TRs. At each TR, blue circles represent the brain areas whose BOLD phase projects into the main BOLD phase direction (captured by the leading eigenvector, see **(C)**, and red dots represent the brain areas whose BOLD phase project into the opposite direction of the main BOLD phase orientation. It serves to illustrate that the phase-shifted signals (in red) do not directly correspond to supra-threshold BOLD increases. **(C)** Phase portraits of the analytic BOLD signal at each TR, where the real and imaginary axis represent the cosines and sines of the Hilbert phase at each TR. **(D)** Representation of the brain patterns captured by the signs of the leading eigenvector at each TR, illustrating how phase-locking patterns evolve smoothly over several TRs, whereas the corresponding BOLD signals (shown in panel **B**) exhibit significantly different activation patterns over the same range of TRs. **(E)** Representation of the instantaneous phase coherence matrices obtained at each TR as the cosine of the phase difference at each instant of time.

To reduce the dimensionality of the voxel-based data (Voxels × Time), the Anatomic Automatic Labeling (AAL) atlas was used to define *N* = 90 anatomically distinct cortical and sub-cortical regions covering the whole brain, excluding the cerebellum. Data was reduced to size N × Time, with Time = 1200 TR per session, by averaging the BOLD signals in all the voxels associated to each brain region (Figure 1A).

### Analysis

#### BOLD Phase Dynamics

To compute the phase relationship between brain regions, for each region *n* with *n* = 1…*N*, a BOLD phase θ(*n*,*t*) varying in time *t*, was calculated via Hilbert transform (Glerean et al., 2012). The analytical signal expresses the regional signal *x*(*t*) as *x*(*t*) = *A*(*t*)*θ(*t*)) with *A* and θ representing the time-varying amplitude and phase respectively (Figure 1C). The first and last time points were removed from each time series, to exclude the boundary artifacts induced by the Hilbert transform. Subsequently, for every pair of brain regions *n* and *m* at time *t* the phase coherence matrix *dPC* is calculated as follows: *dPC*(*n*,*m*,*t*) = *cos*(θ(*n*,*t*)−θ(*m*,*t*)), where *cos*(0) = 1 represents the case when the two brain areas *n* and *m* are aligned at time *t* (Figure 1E). Conversely *cos*(π) = −1 indicates the two brain areas *nandm* to be anti-aligned at time *t*. Lastly, *cos*(π/2) = 0 shows the two brain areas *n* and *m* at time *t* to be orthogonal to each other and therefore their phase relationship being 0.

#### Phase Dynamics Leading Eigenvector

We used LEiDA, where only the *1xN* leading eigenvector *V*_1_(*t*) of the *dPC* is considered in the analysis, to describe the phase coherence pattern of the (*NxN) dPC(t)* at every time-point *t* with reduced dimensionality. In other words, we calculated the eigendecomposition of dPC(*t)* at every time *t* [*dPC*(*t*) = *V*(*t*)*D*(*t*)*V*^−1^(*t*), where columns of *V*(*t*) are the corresponding eigenvectors of *dPC*(*t*) and *D*(*t*) is the diagonal matrix of the eigenvalues of *dPC*(*t*), and we took the first (most dominant) eigenvector*V*_1_(*t*) to represent the BOLD PL pattern at each time point with size *1xN*. Since *dPC(t)* is symmetric, its eigenvectors are orthogonal (*V*^–1^(*t*) = *V*(*t*)*^*T*^*) and the eigenvalues are real. Each element in the eigenvector can be associated to a specific brain area [i.e., in Figures 1B–D each brain area is colored according to its sign in *V*_1_(*t*)]. The *NxN* dominant connectivity pattern at every time *t* captured by *V*_1_(*t*) can simply be retrieved by calculating the matrix product of the eigenvector with its transpose as $V_{1}\left(t\right)*V_{1}^{T}\left(t\right)$ (Cabral et al., 2017b).

**FIGURE 2:**
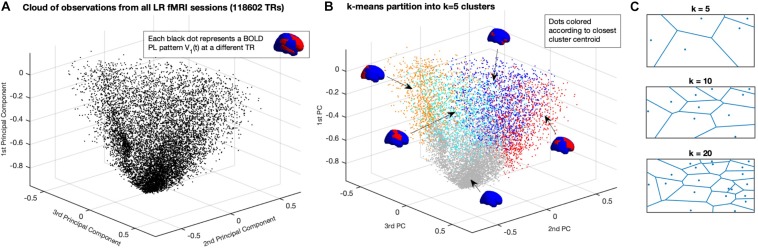
Partition of the *N*-dimensional phase space into a K-dimensional state-based space. **(A)** Representation of all BOLD PL patterns captured at each TR in a reduced 3-dimensional perspective of the phase space. Since each observation is a 1xN vector – corresponding to the leading eigenvector of BOLD phases at each TR – the full phase space is *N*-dimensional, where *N* = 90 is the number of ROIs used to parcellate the brain). Each dot corresponds to one fMRI volume recorded over time (TR = Repetition Time = 0.72 s). Dots are placed according to their cosine distance with respect to the first three principal components (i.e., the first 3 eigenvectors of the covariance matrix) of all observations. **(B)** Partition of the Phase Space using *K*-means clustering decomposes the space of observations into k clusters, where each observation from an fMRI experiment is assigned to a cluster given its closest proximity to the corresponding centroid. The centroids obtained for K = 5 are represented by coloring each brain area using the same color scheme as in Figure 1, representing distinct whole-brain BOLD phase-locking patterns. **(C)** Illustration of the partition of a 2D plane into k Voronoi cells, where each point in a given cell is closer to its centroid than to any other centroid.

With the aforementioned reduction, whole-brain activity at each time point *t* is represented by a *1xN* vector, where *N* is the number of brain regions defined by the applied parcellation. Each vector *V*_1_(*t*) can be seen as an observation of the dynamical system and can be represented as a point in a *N*-dimensional space *R*^*N*^ (in Figure 2A, represented in *R*^3^ for illustration). Each fMRI experiment is thus characterized by a trajectory of the leading eigenvector *V*_1_ in this *N*-dimensional space. To get a graphical representation (Figures 2A,B), we project each vector *V*_1_(*t*) on the space determined by the first three principal components of all *V*_1_s, i.e., the *x*, *y*, and *z* coordinates are given by the cosine distance between each *1xN V_1_*(*t*) and the first 3 *Nx1* eigenvectors of the *NxN* covariance matrix of all *V*_1_s (with size *NxT*).

#### Partition of Phase Space

In order to achieve a state-based representation, the leading eigenvectors obtained from all 99 participants in the Left-Right (LR) fMRI scanning session – corresponding to a total of *T* = 118602 observations (99 × 1198 TRs) with *N* = 90 dimensions each – are partitioned into a set of discrete states. Importantly, we do not include in this partition the Right-Left (RL) fMRI scanning sessions from the same 99 participants recorded on the same day, which will serve to test the validity and consistency of the results, as described in the following section.

Given the large number of observations in this dataset, clustering algorithms relying on the *TxT* similarity matrix had to be discarded because of limited computational resources (i.e., computing our *TxT* matrix requires >100 GB of RAM). Instead, we use the k-means algorithm, which relies on an iterative process to find the solution that minimizes the distance between each (*1xN*) observation and the closest 1xN cluster centroid (we note that, given the large number of dimensions, we use the Cosine distance, which significantly reduces the computation time with respect to City Block or Euclidean distances). As such, *k*-means algorithm is used to iteratively cluster the leading eigenvectors into *k* = 2 to *k* = 20 clusters (resulting in 19 partitions), repeating each calculation 100 times to ensure stability in the results. Since each observation represents a time point, the output vector of cluster assignments – where each observation is assigned to its closest *1xN* cluster centroidα = 1…*k* – can be approached as a trajectory $\underline{x}\left(t\right)$ in state space.

In Figure 2 we show how the *k*-means clustering algorithm divides the phase-space (here represented in only three dimensions for illustration) into *k* = *5* (Voronoi) cells, where each location in the phase space is assigned to the closest centroid. Using colors to represent the regions of the phase space assigned to each cluster *R*^α^, we represent in Figure 2B the same observations from panel A, but highlighting the cluster assignment at each time point. Although the 3-dimensional representation serves to illustrate the partition of the phase-space, there is a clear overlap of colors given that the phase-space, defined in *N* = 90 dimensions, cannot be adequately represented in three dimensions only. To illustrate the decomposition into k-means clustering algorithm we show, in Figure 2C, the partition of a 2D plane into *k* Voronoi cells, where each region in space is assigned to the closest centroid.

Each cluster *R*^α^(with α = 1…*k*) is now represented by its cluster centroid *V*_*c*α_, each corresponding to a distinct BOLD phase-locking state (which will be described in detail in the section “Results”). To assess the quality of the cluster separation, the silhouette value is computed for each *k*, which estimates how similar each observation *V*_1_(*t*) is to its own cluster compared to other clusters.

#### Comparison to Reference Intrinsic Functional Networks

The existence of functionally interconnected subsystems, where subsets of brains areas consistently activate together even during rest, has been widely explored in studies of resting-state functional connectivity. Intrinsic Functional Networks, typically assessed using correlation analysis, have been consistently detected in large cohorts of resting-state fMRI experiments (Yeo et al., 2011), but the analysis of their temporal dynamics has been hindered by the methods used to assess them, namely sliding window methods with their choice of the window over which connectivity is computed (Hindriks et al., 2016).

Here, we verify if the centroids obtained from clustering BOLD phase leading eigenvectors obtained at TR resolution share spatial similarities with the seven cerebral intrinsic functional networks estimated by Yeo et al. (2011) clustering correlation-based functional connectivity between 1175 regions of interest from 1000 participants.

To do so – and since our BOLD PL centroids *V*_α_ are defined in AAL parcellation – we take the mask of the Yeo parcellation into seven non-overlapping functional networks defined in MNI152 space^1^ and the mask of the AAL parcellation in the same MNI152 space, and calculate, for each of the 90 AAL brain areas, the proportion of voxels assigned to each of the seven functional networks, obtaining in this way 7 1 × 90 vectors representing the intrinsic functional networks in AAL space.

Subsequently, we compute the Pearson’s correlation (with associated *p*-values) between these seven networks and the centroids *V*_α_ obtained from our clustering analysis across the whole range of *k* explored (setting all negative values of the centroids’ vectors to zero, to consider only the areas whose BOLD phase is shifted from the main orientation).

#### Projection of the Validation Dataset Into the Same State Space

We used the second fMRI scanning session from each of the 99 participants recorded on the same day as the primary dataset – differing only in the oblique axial acquisition phase encoding, being Right to Left (RL) instead of Left to Right (LR) – to verify the validity and consistency of the partition performed in the previous session. To do so, we obtained all the *1xN* leading eigenvectors from the Right-Left (RL) scanning session – totaling 118602 observations (1198 TRs × 99 subjects) – using the same methodology as before, but instead of running the k-means algorithm, we compute the cosine distance between each *1xN* eigenvector *V*_1_(*t*) and the *k 1xN* cluster centroids *V*_*c*α_ obtained from the previous analysis, and define the trajectory vector $\underline{x}\left(t\right)$ by assigning each *V*_1_(*t*) to its closest cluster centroid *V*_*c*α_.

#### Fractional Occupancy

Following the cluster partition into *k* PL states evolving in time *t*, the probabilities – or fractional occupancies – $\Pi_{\alpha }^{\left(S\right)}$ associated to each PL state α and each scan *S*, can be calculated as follows:

(1) $$\Pi_{\alpha }^{\left(S\right)}=\frac{1}{T}\sum_{t=1}^{T}\chi \left[\underline{x}\left(t\right)\in R^{\alpha }\right]$$

where *χ* is the indicator function – χ(*A*) = 1 if the event *A* is true, and χ(*A*) = 0 otherwise, and *T* = 1198 is the number of time points (TRs) corresponding to each fMRI scan *(S)*. In other words, the equation counts the number of times when the trajectory $\underline{x}\left(t\right)$ is assigned to each of the defined clusters *R*^(α)^, divided by the total number of time points *T*. Furthermore, given that participants are constantly in resting state – i.e. without performing any task -, we assume stationarity in the data within each scan (justifying the time average in Eq. 1). Cluster probabilities are estimated separately for each individual fMRI scan.

#### Dwell Time

To describe the average time periods when a given PL state α is being visited in each fMRI scan *S*, the Dwell Time $DT_{\alpha }^{\left(S\right)}$ is defined as the mean of all the consecutive periods of each state, i.e.,

(2) $$DT_{\alpha }^{\left(S\right)}=\frac{1}{p_{\alpha }}\sum_{1}^{p_{\alpha }}C_{p_{\alpha }}$$

where *DT*_α_ is the Dwell Time of PL state α, *p*_α_ is the number of consecutive periods assigned to PL state α and *C*_*p*_α__ is the duration of each consecutive period.

#### Markov Chain Transition Probabilities

Following the same rational as in Eq.1, the definition of the probability Π_αβ_ to be in the PL state α at time bin *t* and in the PL state β at time bin t + 1 can be written as follows:

(3) $$\Pi_{\alpha \beta }^{\left(S\right)}=\frac{1}{T-1}\sum_{t=1}^{T-1}\chi \left[\underline{x}\left(t\right)\in R^{\alpha },\underline{x}\left(t+1\right)\in R^{\beta }\right]$$

and thus, the transition probability matrix $W_{\alpha \beta }^{\left(S\right)}$ of each fMRI scan *S* is defined as:

(4) $$W_{\alpha \beta }^{\left(S\right)}=P\left[\underline{x}\left(t+1\right)\in R^{\beta }|\underline{x}\left(t\right)\in R^{\alpha }\right]=\frac{\Pi_{\alpha \beta }^{\left(S\right)}}{\Pi_{\alpha }^{\left(S\right)}}.$$

*W*_αβ_ defines the transition matrix from state alpha to state beta. This defines an homogeneous Markov chain, characterizing the transition between BOLD phase locking states. The transition probability matrix $W_{\alpha \beta }^{\left(S\right)}$ is estimated separately for each scan *S*. To each matrix $W_{\alpha \beta }^{\left(S\right)}$ is associated a transition graph with an oriented arrow from α to β if $W_{\alpha \beta }^{\left(S\right)}$ > 0 (see Supplementary Figures S2–S4). To illustrate the transitions at the group level, we represent the transition graph of the average transition matrix *W*_αβ_ across all scans in the LR sessions.

#### Intra-Class Correlation

In order to calculate the reliability of the computed measures between the LR and RL fMRI sessions recorded on the same day, we calculated the Inter-Class Correlation (ICC) (Landis and Koch, 1977; Xing and Zuo, 2018). ICC describes the proportion of within-subject variability versus between-subject variability across recording conditions as follows:

(5) $$ICC=\frac{MSE_{b}-MSE_{w}}{MSE_{b}+MSE_{w}},$$

where *MSE*_*w*_ and *MSE*_*b*_ are the within-subject and between-subject mean squared errors, respectively (Xing and Zuo, 2018). Positive ICC values (i.e., when within-subject MSE is smaller than the between-subject MSE) indicate individual reliability, which, depending on its value, is categorized as low (0 < ICC < 0.2), fair (0.2 < ICC < 0.4), moderate (0.4 < ICC < 0.6), substantial (0.6 < ICC < 0.8) and almost perfect (0.8 < ICC < 1) (Landis and Koch, 1977).

#### Effects of Low-Pass Temporal Filtering

A typical step in the pre-procession of fMRI resting-state data is the application of a low-pass filter to exclude high frequency noise in the BOLD signal (typically < 0.1 Hz). However, given that BOLD signals are already averaged over all voxels within each brain area – which should improve the signal-to-noise ratio-, and given the instantaneous nature of our dynamic analysis, we performed our first analysis directly on the unfiltered BOLD signals recorded at a TR of 0.72 s, corresponding to a Nyquist frequency of fNq = 1/2TR = 0.694 Hz.

To verify whether the higher frequency components in the BOLD signal are meaningful for the dynamic analysis of functional networks, we apply a 2nd order Butterworth band-pass filter to the ROI-averaged BOLD signals – before computing LEiDA and clustering into *k* = 5 states – varying the low-pass cut-off frequency to 0.07, 0.1, 0.2 or 0.6944 Hz, while keeping the lower high-pass frequency limit at 0.01 Hz (to exclude only the ultra-slow signal drifts from the scanner). ICC measures were subsequently calculated for the corresponding Dwell Times, Fractional Occupancy and Transition Probabilities obtained.

## Results

### Phase-Locking States Reveal Relevant Functional Networks

We obtain a set of BOLD phase-locking patterns from the first session of resting-state fMRI of 99 unrelated subjects using the LEiDA approach (see section “Materials and Methods” for details). Each BOLD phase-locking pattern is represented as a vector with *N* elements, each element representing the projection of the BOLD phase of a brain area into the leading eigenvector of all BOLD phases (here *N* = 90 since we use the 90 non-cerebellar brain regions from the AAL atlas).

**FIGURE 3:**
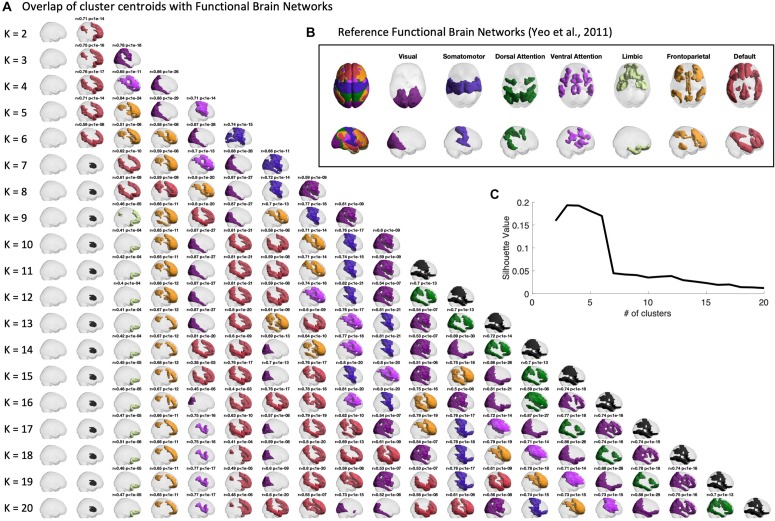
Centroids overlap with Functional Brain Networks. **(A)** Representation of the centroids obtained for each k-means clustering solution with K ranging between 2 and 20. Centroids are represented in cortical space, rendering only the ROIs whose BOLD phase is shifted > ±π/2 with respect to the leading direction. ROIs are colored according to the reference Functional Brain Network (shown in panel **B**) to which they most significantly relate with. Pearson’s r and corresponding *p*-value are reported as a title only when surviving a conservative threshold of *p* < 0.05/K, to correct for the number of independent hypotheses tested in each partition model. Centroids not significantly overlapping with any of the reference functional networks are colored in black. Side views of the same centroids are reported in Supplementary Figure S1. **(B)** Reference functional brain networks estimated from 1000 subjects from correlation-based intrinsic functional connectivity (Yeo et al., 2011). **(C)** Silhouette value, used to evaluate clustering performance, shows a peak for partitions into 2 to 6 clusters.

Firstly, we verify the overlap between the BOLD phase-locking states obtained across clustering solutions (with 2 < *k* < 20) to seven intrinsic functional networks defined in the literature (Yeo et al., 2011). In Figure 3, we report for all partitions into *k* states (rows), the *k* cluster centroids obtained (columns). The cluster centroids *V*_*c*α_(representing BOLD phase-locking states) are represented in the brain by coloring only the brain areas whose BOLD phase projects in the opposite direction from the main orientation of BOLD phases (negative elements in *V*_*c*α_). BOLD phase locking states are color-coded according to the most significantly correlated RSN used as reference (shown in panel B), given a corrected threshold of *p* < 0.05/*k*, and in black otherwise. The same Figure is shown from a top view perspective in Supplementary Figure S1.

Sorting the states according to their probability of occurrence, we find consistently across clustering solutions a most prevalent state, occupying the first column of Figure 3, in which the BOLD phase of all brain areas project into the same direction. Since it does not reveal the separation of any particular subsystem, and does not significantly overlap with any reference functional network, this so-called global state (state 1) is represented as a transparent brain.

The remaining states are all characterized by a phase shift in the BOLD signal of a given subset of brain areas, which are highlighted as colored patches. Notably, most of the obtained cluster centroids demonstrate a close statistical similarity to reference functional networks, revealing a strong and highly significant overlap (up to *r* = 0.89, with *p*-values down to10^−30^) with the different RSNs used as reference. We also find that some partitions show different PL states overlapping with the same reference RSN. When no significant overlap is found, the patches are colored in black. One example is the second most prominent state appearing in all clustering solutions with *k* > 7, which involves regions of basal ganglia, which have been omitted in the analysis of functional networks from Yeo et al. (2011).

We chose to focus on the clustering solution with *k* = 5 within the range of best clustering performance according to the silhouette value (Figure 3C), as it reveals a meaningful partition of the BOLD PL patterns into four representative functional networks. For *k* = 5 we found State 2 to correlate with the Default Mode Network (*r* = 0.71, *p* = 10^–14^), State 3 to correlate with the Fronto-parietal Network (*r* = 0.84, *p* = 10^–21^), State 4 to correlate with the Visual Network (*r* = 0.88, *p* = 10^–29^) and finally State 5 to mostly correlate with the Ventral Attention Network (*r* = 0.71, *p* = 10^–14^). In Supplementary Figures S5, S6, we also report the overlap of cluster centroids with reference functional brain networks obtained for the filtered series (0.04–0.07 Hz) both from top and side view.

### Exploration of a Repertoire of BOLD Phase Locking States

**FIGURE 4:**
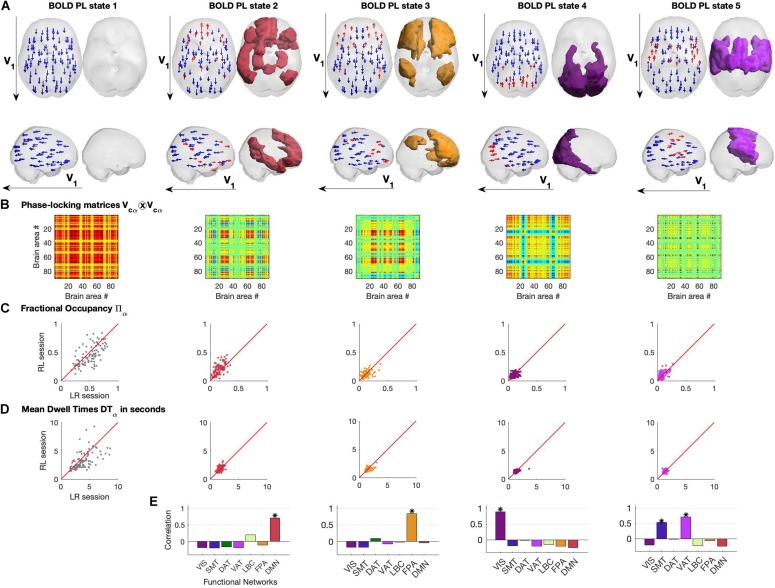
Repertoire of BOLD Phase Locking States obtained using *K* = 5. **(A)** BOLD Phase Locking states represent recurrent patterns of BOLD phase alignment across the whole brain. Each centroid is a vector V_*C*_ of size 1xN whose elements indicate how each brain area projects into it. Each centroid is represented in the brain in two different ways: (left) by placing an arrow at the centre of gravity of each brain area and setting its size, direction and color according to the magnitude and sign of the corresponding element in V_*C*_ (coloring in red for positive projections into V_*C*_, and blue otherwise). (right) Rendering all brain areas with positive values in V_*C*_ colored according to the functional network to which they show maximal overlap (see Figure 3 and/or panel **E** below). **(B)** Phase-locking matrices computed as the outer product of the centroid vectors V_*C*_. **(C)** Scatter plots of state Fractional Occupancy, plotting the values obtained for the 99 fMRI scans in the LR scanning session versus the values from the RL session. **(D)** Scatter plots of mean state Dwell Times, plotting the values obtained for the 99 fMRI scans in the LR scanning session versus the values from the RL session. **(E)** Correlation between each BOLD PL state and the seven networks of intrinsic functional connectivity from Yeo et al. (2011). * indicates significance corrected for multiple comparisons with Pearson’s *p*-value < 0.05/7.

In Figure 4, we show the different representations of the BOLD PL states and their properties. Each PL state is represented in two ways: on the left we plot the *N* = 90 vector elements as arrows representing the magnitude of projection of each brain area into the leading eigenvector of BOLD phases *V*_1_ and on the right by rendering and coloring the brain regions shifted from the main orientation (corresponding to the red arrows on the left) according to the relevant functional system to which they maximally overlap with (Figures 4A,E).

The PL states can also be represented in the form of a matrix by computing the matrix product of each centroid’s vector *V*_*c*α_ and its transpose, describing the pairwise relationship between individual brain regions in each PL state (Figure 4B).

Assuming stationarity of the brain’s dynamical regime during rest, we compute the probability of occurrence of PL states as well as their mean Dwell Time within each fMRI scan (see section “Materials and Methods”). In Figures 4C,D, we show the probabilities of occurrence and mean Dwell Times of each PL state obtained for each participant, plotting the values obtained from the first fMRI session (LR) versus the values obtained from the second same-day fMRI session (RL). We find that, in both LR and RL sessions, State 1 shows high variability both in terms of probability of occurrence (mean = 0.51, standard deviation (std) = 0.16) and Dwell Times (mean = 3.94 s, std = 1.73 s), with some subjects spending as little as 20% of the time in this globally coherent state, whereas others spend up to 80% of the time, with some occurrences lasting up to 10 s (the reliability of metrics across recordings will be addressed in a following section). Interestingly, the other 4 states show consistently lower probabilities of occurrence, with state 2 (overlapping with the DMN) occurring on average 16.6 ± 7.6% of the time (mean ± std), being slightly more prevalent than the other states (state 3: 12.7 ± 6.2%; state 4: 9.9 ± 4.7%; state 5: 9.5 ± 5.5%). Not only do these functionally relevant PL patterns occur less often, but they also show, consistently across subjects, much shorter Dwell Times, lasting on average around 2 TRs (state 2, 1.71 ± 0.34 s; state 3 1.57 ± 0.37 s; state 4, 1.4 ± 0.34 s; state 5, 1.3 ± 0.22 s).

In Figure 4E, we report the correlation between each PL state and the seven intrinsic functional networks used as reference (see section “Materials and Methods”). We observed State 2 to correlate only with the Default Mode Network (*r* = 0.71, *p* = 10^–14^), State 3 to correlate with the Fronto-parietal Network (*r* = 0.84, *p* = 10^–24^), State 4 to correlate with the Visual Network (*r* = 0.88, *p* = 10^–29^) and finally State 5 to mostly correlate with the Ventral Attention Network (*r* = 0.71, *p* = 10^–14^) but also with the Somatomotor Network (*r* = 0.53, *p* = 10^–8^). Supplementary Figure S7 of the states’ measures for the filtered data (0.04–0.07 Hz) is added in the Supplementary Material.

### Recurrent Excursions Into BOLD PL States

**FIGURE 5:**
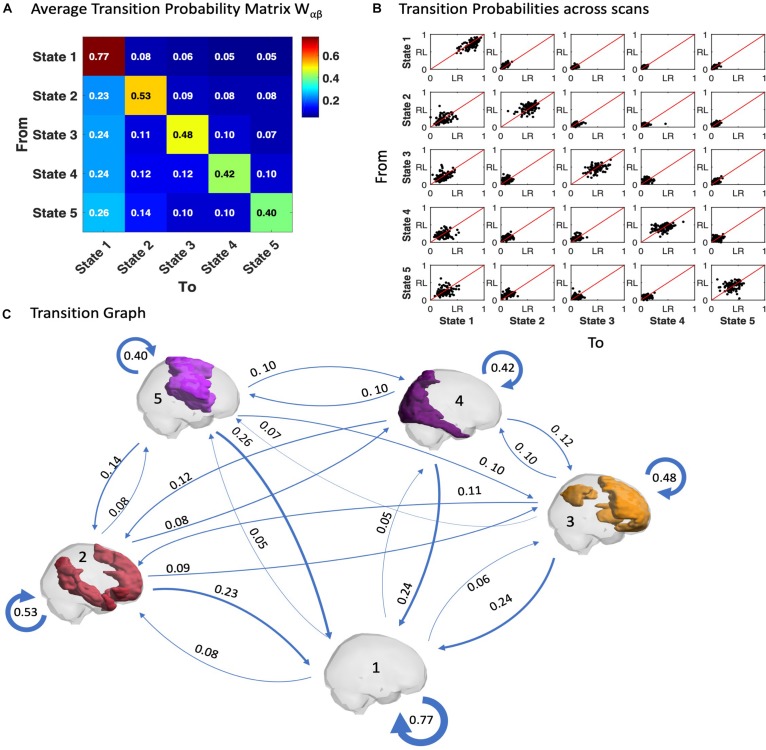
Trajectories of brain activity in state space. **(A)** Transition matrix quantifying the probability of the trajectory transiting from one state to another as defined in Equation 5, averaged across the scans from all 99 participants in the LR session. **(B)** Transition probabilities estimated for each fMRI scan, each dot corresponding to one participant, plotting the probabilities of switching in the LR fMRI session versus the RL fMRI session. **(C)** Transition Graph is constructed from transition matrix *W* where edges α→β are directed and weighted with weight *W*_αβ_.

Similar to the probability of occurrence of a given state, we can quantitatively characterize the temporal trajectories by the probabilities of transition between the different BOLD PL states. In Figure 5A we show the average transition matrix, *W*_αβ_, as the probability of switching from state α to state β. We noted that the highest probabilities of transition (*W*_αβ_ > 0.5) were along the diagonal (representing the probability to remain in the same state) as well as along the first column (representing the transitions back to the state 1). The characteristic self-transitions (α → α) along the diagonal are a distinctive feature of the system, indicating the relative stability of each state. State 1 reveals the highest stability (with 77% probability of remaining in it in the following TR), whereas the probability of remaining in the other states is close to chance levels. The scatter plots in Figure 5B show the transition probabilities obtained for each of the 99 participants (LR session vs. RL session), revealing consistency of the results across participants and scanning sessions.

Another relevant feature is the asymmetry of the transition matrix, which is indicative of an imbalance in the reciprocity of transitions both to and from a given state, as can be observed with the apparent proclivity for switching into the (global) state 1, whereas the probability to leave from it is much smaller.

In Figure 5C, a Transition Graph is constructed from the transition matrix *W* shown in panel A, where edges α → β are directed and weighted with weight *W*_αβ_. This gives a good insight into the spontaneous transition dynamics and motivates the use of the Markov chain transition matrix beyond the probability of occurrence alone. Supplementary Figure S8 of the transition graph and matrix for the filtered data (0.04–0.07) is added in the Supplementary Material. Furthermore, the comparison of LR and RL sessions for the probabilities of transition is added in Supplementary Figure S10.

### Reliability of Individual Metrics

**FIGURE 6:**
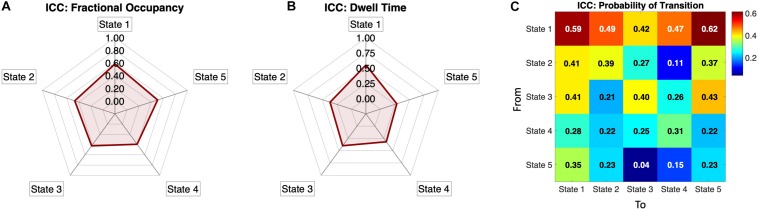
Individual reliability of the Phase-locking states’ measures: **(A)** Intra-Class Correlation (ICC) calculated for the Fractional Occupancy, showing positive ICC values for all five PL states, meaning that the within-subject error is smaller than the between-subject error. All values are within a moderate range of within-subject reliability (i.e., 0.4 < ICC < 0.6) according to the categorization by Landis and Koch (1977). **(B)** Intra-Class Correlation for Dwell Times for all five states showing the states 1,2 and 3 to be in the moderate reliability range. **(C)** ICC for the normalized Probability Transition Matrix showing positive ICC values in all transitions, with the probabilities of transition from state 1 being the most reliable, whereas other transitions, particularly between states 2 to 5, showing lower reliability. The ICC is categorized, based on (Landis and Koch, 1977) as low (0 < ICC < 0.2), fair (0.2 < ICC < 0.4), moderate (0.4 < ICC < 0.6), substantial (0.6 < ICC < 0.8) and almost perfect (0.8 < ICC < 1).

To assess the metric’s reliability across same-subject same-day recordings when compared to other subjects, we computed the Intra-Class Correlation (see section “Materials and Methods”) for each measure above, namely, the Fractional Occupancies, the Dwell Times and the Transition Probabilities. In Figure 6A, we show the Fractional Occupancy for all 5 states to have moderate reliability values (State1: ICC = 0.59, State 2: ICC = 0.47, State 3: ICC = 0.42, State 4: ICC = 0.39 and State 5: ICC = 0.51). The Dwell Times for the first three states had moderate values of ICC and States 4 and 5 showed poor values (State1: ICC = 0.55, State 2: ICC = 0.37, State 3: ICC = 0.4, State 4: ICC = 0.32, and State 5: ICC = 0.28, Figure 6B).

Regarding the transition matrix, Figure 6C shows that the probability of remaining in the (global) State 1 has one of the highest ICC values (ICC = 0.61), with most of the transition to and from the State 1 showing a range of fair to moderate ICC values (0.29 < ICC < 0.62). States 2 and 3 have border-line moderate values of ICC in the self-transitions (State 2: ICC = 0.39, State 3: ICC = 0.40) and some of the transitions to other states were also in the moderate range. States 4 and 5 seem to have relatively poor, but still positive, ICC values for the probability of transitions metric (Figure 6C).

Taken overall, the ICC results show that all the measures evaluated have smaller within-subject error than the between-subject error (given ICC values are positive for all measures), indicating that the measures proposed herein capture individual fingerprints of dynamic functional connectivity. To improve the assertion of individual landscapes and reliability of the methodology we added scatter plots for all the three measures (Fractional Occupancy, Dwell Times and Transition Probabilities) of the two sessions (LR and RL) in Figures 4, 5.

### Effect of the Temporal Filtering

All the results shown so far were obtained directly from the ROI-averaged BOLD signals from the HCP dataset, without applying any temporal frequency filter. Temporal filtering is a typical pre-processing step in resting-state fMRI analysis to remove frequency components regarded as noise. In this section, we evaluate whether the inclusion of the higher frequency components in the BOLD signal improve the analysis of dynamic functional connectivity by evaluating its effects on the reliability (ICC) of the measures across sessions, which should be maximized if assuming stationarity in individual resting-state dynamics.

**FIGURE 7:**
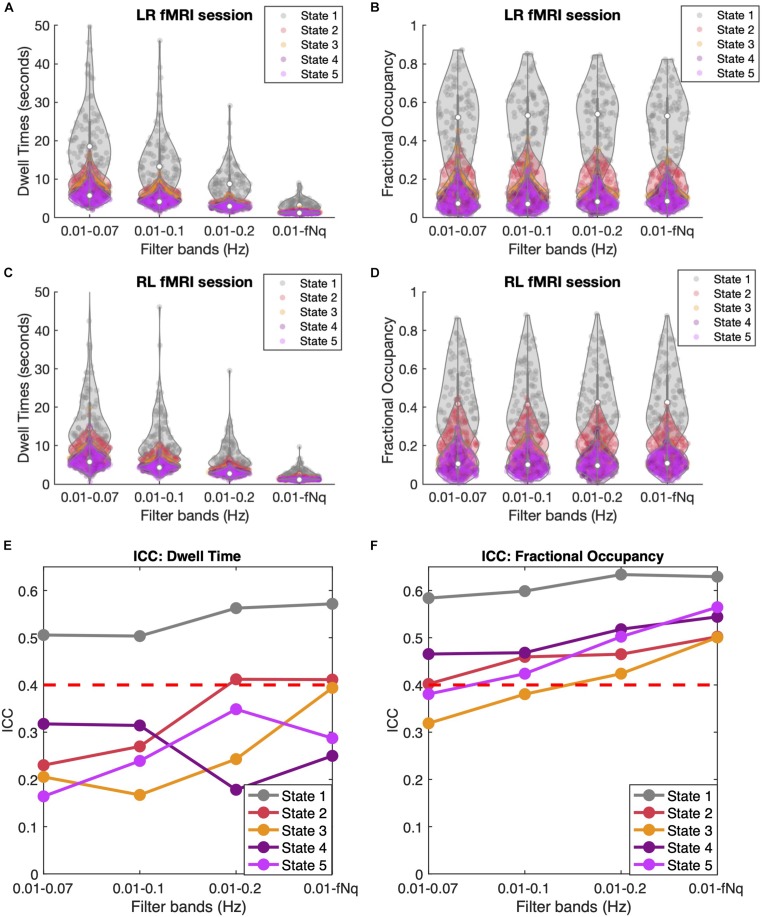
Temporal Filtering effect on Dwell Time and Fractional Occupancy. **(A,C)** Dwell Times obtained across four different band-pass filters applied to the BOLD signals, keeping the lower bound fixed at 0.01 Hz and varying the higher (lowpass) cutoff frequency between 0.07, 0.1, 0.2, and 0.6944 Hz, which corresponds to the Nyquist frequency fNq = 1/(2TR). Results are shown for LR **(A)** and RL **(C)** recording sessions. **(B,D)** Fractional Occupancies across the four different filtrations for LR **(B)** and RL **(D)** recording sessions. **(E,F)** Inter-Class Correlation (ICC) across four different filtrations for Dwell Times **(E)** and Fractional Occupancy **(F)**. The red dashed line represents the threshold for moderate reliability based on the Landis and Koch (1977) ICC scale.

As shown in Figures 7A,C, filtering has a crucial effect on the Dwell Times, with lower cut-off frequencies leading to longer Dwell Times for all states, and especially for state 1. Notably, when reaching up to the Nyquist frequency, the mean Dwell Times of states 2 to 5 approach the duration of 2TRs consistently for all subjects and in both LR and RL fMRI sessions, while state 1 lasts for slightly longer periods. When evaluating the Dwell Times ICC (Figure 7E), we find that the ICC is maximal for states 1, 2, and 3 when the high frequency components of the BOLD signal are included. However, it is important to take into account that the accurate estimation of Dwell Times is limited by the temporal resolution of the current fMRI dataset, minimizing the difference between subjects and hence affecting the ICC estimation.

Regarding the Fractional Occupancy of the states, it appears from Figures 7B,D that filtering does not affect the overall values estimated across subjects and in the different sessions. However, when looking at the ICC values for Fractional Occupancy (Figure 7F), we find that this measure is much more reliable within individuals (and across all states) when the high frequency components of the BOLD signal are included. Similarly, the ICC of the Transition Probabilities (shown in Supplementary Figure S9) shows a slight improvement in the reliability of most transitions. Actually, when considering only frequencies <0.2 Hz, a few transition probabilities have even a negative ICC, indicating no individual reliability at all. Overall, these findings point to the direction that it is important to consider the high frequency components of resting-state BOLD signals when assessing individual fingerprints in dynamic functional connectivity.

## Discussion

The challenge of describing dynamic functional connectivity for a mechanistic understanding of the brain processing as well as for its potential use in clinical research, has been of great interest to the neuroimaging community (Hutchison et al., 2013; Preti et al., 2016). With the advent of open multimodal neuroimaging data, it is possible to address and validate these approaches in representative datasets (Van Essen et al., 2013; Poldrack and Gorgolewski, 2014). In this work, we apply, formalize and validate the Leading Eigenvector Dynamics Analysis (LEiDA) to a large cohort of 99 healthy unrelated HCP subjects (Glasser et al., 2013). We describe brain activity during rest as a time evolving trajectory in a low-dimensional state space, where states are defined according to characteristic whole-brain configurations of BOLD phase-locking. Furthermore, we validate these BOLD phase-locking states to reference networks of intrinsic functional connectivity (Yeo et al., 2011) and compute their properties of fractional occupancy, Dwell Times and transition probabilities. We subsequently assess the reliability of these measures across the two same-day fMRI recordings (using Intra-Class Correlation) and show that all measures have a smaller within-subject error than the between-subject error (ICC values > 0), with the highest reliability values being detected when including the high frequency components (>0.1 Hz) of ROI-averaged BOLD signals in the analysis. We argue that such interpretation of brain activity, validated with reliability analysis, has the potential to identify individual-specific fingerprints in the brain’s dynamical landscape and thus serve personalized clinical applications in diagnostics and therapeutics of patients with cognitive disorders.

Concepts and methods from dynamical systems theory are proving useful in the analysis of brain activity at the macroscopic scale, as they serve to formally characterize the complex dynamics emerging from the collective behavior of billions of interacting neurons, exhibiting features such as multi-stability, meta-stability and self-organized criticality, that may serve helpful to identify the underlying principles coordinating cognition at the whole-brain level (Deco and Jirsa, 2012; Tognoli and Kelso, 2014; Cocchi et al., 2017; Roberts et al., 2019). Here, we aimed at a different characterization of the dynamical properties of the intrinsic functional networks emerging spontaneously and consistently during rest. Our analysis revealed a repertoire of BOLD phase-locking states through which the trajectory of brain activity consistently returns in time and across subjects.

By analyzing the PL state’s fractional occupancy, Dwell Time and probability of transitions, our results revealed that the BOLD phase-locking states can be divided in two groups according to their dynamical properties: On one hand, our algorithm consistently detects a state where all the ROI-averaged BOLD signals project into the same direction captured by the leading eigenvector (state 1 for all clustering solutions). This state exhibits longer Dwell Times and shows high between-subject variability but also high within-subject reliability. On the other hand, we detect for all k-means clustering solutions, a set of k-1 states where the BOLD signals of some brain areas project into the opposite direction from the main BOLD phase orientation. These states occur consistently less often and last for shorter times than the global state, but reoccur consistently across subjects and sessions. Given the reduced stability of these BOLD phase-locking states with respect to the meta-stable globally synchronized state, we refer to this second group as “ghost” attractor states. In other words, ghost attractors in this framework refer to short-lived (or weakly stable) network configurations that consistently reoccur across fMRI recordings.

Regarding the functional relevance of these “ghost” phase-locking states, our results show a clear and highly significant overlap of most cluster centroids (obtained for the whole range of partitions explored) with a set of seven previously identified networks of intrinsic functional connectivity used as reference. This finding indicates that these patterns of BOLD phase locking, despite being obtained from a different analytic perspective than more conventional correlation-based analyzes, are closely related to the so-called resting-state networks. Yet, unlike correlation-based analyses that reveal only the spatial map of these functional networks, the LEiDA approach allows characterizing their properties over the temporal dimension. As the reference RSNs are computed from the correlation-based static functional connectivity, a perfect match to the BOLD phase-locking states detected herein is not expected. Rather, they can be considered for validation of the functional relevance of the PL states and served to guide the choice of the number of states or further analysis. The number of states chosen is a trade-off between more fine-grained but less robust state solutions as demonstrated by the increasing specificity of functional subsystems for higher k. Here, the clustering solution with *k* = 5 was chosen for being within the range of maximal Silhouette value and for revealing a separation into distinct functionally meaningful systems such as the Default Mode Network, the Frontoparietal Network, the Ventral Attention Network and the Visual Network. However, a partition into a higher number of states may prove necessary when addressing particular conditions that affects a particular subsystem optimally defined for higher *k*. For instance, in a previous work using LEiDA, the partition into *k* = 10 was chosen for detecting the network that most significantly distinguished patients in remission from major depressive disorder and controls (Figueroa et al., 2019), whereas another study found the solution with *k* = 7 to optimally highlight the effects of psilocybin (Lord et al., 2019).

For all partitions into *k* > 7, our algorithm consistently detected a functional subsystem involving the basal ganglia (colored in black in Figure 3 and Supplementary Figure S1) for not overlapping with any of the reference RSNs) as the second most prevalent BOLD PL state. This indicates that resting-state activity also involves connectivity to subcortical areas, which appears particularly important for the study of psychiatric disorders, such as anxiety-related disorders involving the basal ganglia. Following previous LEiDA studies, we chose here a coarse parcellation into N = 90 brain areas and did not include the BOLD signal detected in the cerebellum. The Anatomic Automatic Labeling Atlas has been validated in many studies and has shown consistency in the LEiDA results across datasets (Cabral et al., 2017b; Figueroa et al., 2019; Lord et al., 2019). However, it is based on an anatomic definition of the brain regions and as such might not generalize adequately to the dynamic functional connectivity analysis. We expect to extend to finer-grained and fMRI-derived parcellations in future studies, potentially including other substructures such as the cerebellum, in order to gain a wider insight into the network configurations observed in brain activity at the macroscopic scale (Cammoun et al., 2012; Glasser et al., 2016; Schaefer et al., 2018).

The mechanistic interpretation of the empirical data proposed herein serves as a great candidate for further theoretical exploration by whole-brain computational models (Ghosh et al., 2008; Deco et al., 2009; Honey et al., 2009). To this date, many models have demonstrated well-matched dynamics to the brain activity as represented by static functional connectivity in a critical range of parameters where the brain is poised between noisy and oscillatory activity. Furthermore, different properties were shown to have an impact on the emerging dynamics such as propagation delays, local vs. global connections, signal-to-noise ratio, and local inhibitory rules (Deco et al., 2009, 2014; Honey et al., 2009; Cabral et al., 2011). Extending such modeling endeavors away from static functional connectivity to a dynamic representation of the experimental data is currently becoming a possible new avenue into understanding the underlying principles governing dynamic functional connectivity (Hansen et al., 2015; Cabral et al., 2017a; Deco et al., 2018, 2019). Recently, Deco et al. have shown how the dynamic representation of resting-state data in wakefulness and sleep (characterized using LEiDA) can serve to explore how a whole-brain model can be perturbed to identify the brain regions responsible for the transition between awake and sleep state (Deco et al., 2019).

Representing dynamic functional connectivity through the prism of dynamical system theory hypothesizes the existence of attractors in *N*-dimensional space through which the functional activity evolves in time. Assuming this hypothetical scenario, it describes a state-based propagation of the data, rendering the underlying dynamics in a discrete sense (Baker et al., 2014; Karahanoğlu and Van De Ville, 2015; Preti et al., 2016; Cabral et al., 2017a). However, other methods have considered dynamic functional connectivity from a continuous point-of-view, such as the spatio-temporal connectome where brain activity is described as a temporal graph (Griffa et al., 2017; Vohryzek et al., 2019) and auto-regressive models (Liégeois et al., 2017). We acknowledge that looking at the brain activity in a discrete sense is only one of the interpretations currently proposed in trying to describe the emergent complex phenomena observed in whole-brain dynamics.

It is to be noted that the applied clustering algorithm is just one amongst many decomposition methods that can partition the LEiDA results into meaningful states. Indeed (Cabral et al., 2017b) compared the results from k-means algorithm to the Hidden Markov Model (HMM), in their paper on cognitive performance of patients, showing similar results with both approaches (Cabral et al., 2017b). However, k-means was chosen here for its relatively simple implementation and its relatively low computational cost, revealing functionally meaningful cluster centroids.

New imaging methods benefit greatly from the reliability analysis that investigates individual variabilities across recordings sessions. Especially in clinical applications, reliability is crucial to obtain stable measures across time for individual subjects (i.e., low within-subject variability) and at the same time distinguishable differences between subjects (i.e., high between-subject variability) (Xing and Zuo, 2018; Zuo et al., 2019). In this work, Intra-Class Correlation is used to calculate the desirable ratio between between-subject variability and within-subject variability across recording sessions. One of the Intra-Class Correlation scales proposed by Landis and Koch (1977) to assess reliability for clinical applications suggests that values 1.0 > ICC > 0.8 have excellent reliability, 0.8 > ICC > 0.6 substantial reliability, 0.6 > ICC > 0.4 have moderate reliability and, 0.4 > ICC > 0.2 poor substantial reliability. In other words, it is desirable to obtain high reliability values for the method’s possible clinical application.

In the last part of our study, we show that including the high frequency components up to the Nyquist frequency maximizes the ICC values (reaching a mainly moderate range of ICC values). As such, it is likely that the temporal resolution of the fMRI acquisition might have hindered further increase in reliability. Although the Dwell Times become significantly shorter if no smoothing is applied – which may decrease the detection of RSNs in correlation-based analysis – we find that the occurrence of these states is intrinsically short, given that the measures become more reliable. Although the hemodynamic response function (HRF) is intrinsically slow, the capacity of the BOLD signal to detect faster frequency components is still highly debated in the literature (Glerean et al., 2012; Deco et al., 2019). Nevertheless, it is likely that resting-state dynamics occurs at a faster time scale than captured with the BOLD signal, as suggested by MEG studies that point to a duration of around 200 ms (Baker et al., 2014; Vidaurre et al., 2016). Here, we show that LEiDA allows detecting meaningful dynamic network configurations occurring at relatively short time-scales for fMRI analysis, which may serve useful not only for resting-state analysis but also for the detection of task related patterns [as in Stark et al. (2019)], that may not be captured with conventional general linear models using the HRF. Overall, we expect that novel insights into BOLD signal temporal characteristics and improvements in fMRI temporal resolution might increase the ICC reliability of these measures.

## Conclusion

In summary, we combine novel analytic tools to quantitatively characterize brain activity in each fMRI scan as a trajectory through a discrete set of BOLD phase-locking states. Given the dynamical properties of these states (fractional occupancy, Dwell Time and transition probability) we propose that RSNs behave as ghost attractors, emerging spontaneously and for brief periods, but recurring consistently across subjects and sessions. Our study corroborates previous theoretical works that put forward an interpretation of brain activity as a trajectory evolving in time in an energy landscape (Deco et al., 2011; Ashourvan et al., 2017). By demonstrating the functional relevance of the BOLD phase-locking states detected and the reliability of the measures across same-subject sessions we go further by revealing the existence of individual-specific energy landscapes in brain activity with potential application in patient-specific diagnostics and therapeutics.

## Data Availability Statement

All data and codes used in this study are publicly available on https://github.com/jvohryzek/GhostAttractors.

## Ethics Statement

The studies involving human participants were reviewed and approved by The Washington University review board including all study protocols. Informed consent was obtained for all participants. The participants provided their written informed consent to participate in this study.

## Author Contributions

JV and JC carried out the analysis, wrote the main manuscript and came up with the paper’s ideas. BC and GD verified and advised the theoretical methods. MK and JC supervised the whole project. All authors participated in the discussion of the ideas and contributed in the final writing of the manuscript.

## Conflict of Interest

The authors declare that the research was conducted in the absence of any commercial or financial relationships that could be construed as a potential conflict of interest.

## Footnotes

1 surfer.nmr.mgh.harvard.edu/fswiki/CorticalParcellation_Yeo2011
