# Supplementary material for: Ghost Attractors in Spontaneous Brain Activity: Recurrent Excursions Into Functionally-Relevant BOLD Phase-Locking States
Source: Front Syst Neurosci. 2020 Apr 17;14:20. doi: 10.3389/fnsys.2020.00020 (PMC7182014; doi:10.3389/fnsys.2020.00020)
Supplement: Supplementary file 1 [file Data_Sheet_1.pdf]

frontiers

Supplementary Material

Ghost attractors in spontaneous brain activity: recurrent excursions into a repertoire of functionally relevant BOLD phase-locked states

Jakub Vohryzek, Gustavo Deco, Bruno Cessac, Morten Kringelbach and Joana Cabral

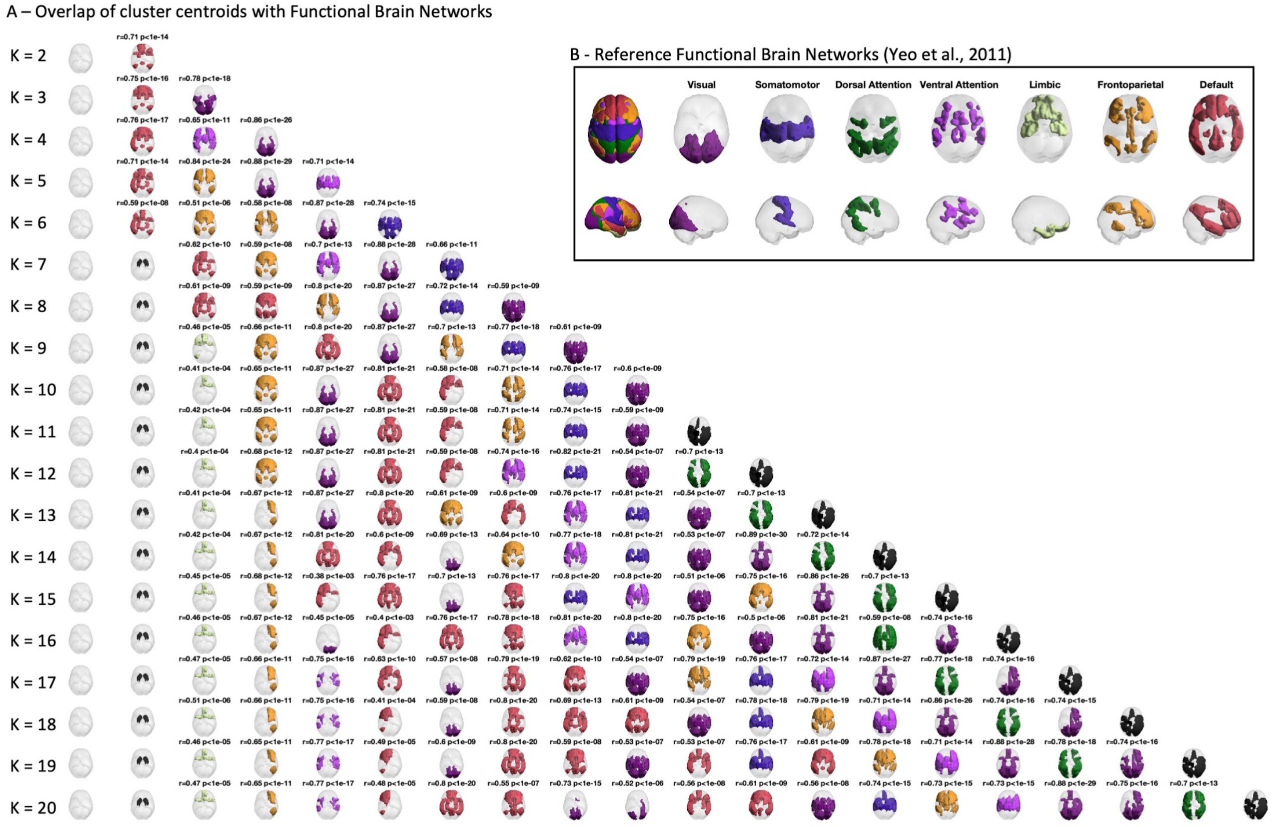

SI Figure 1 - **Overlap of Cluster Centroids with reference Functional Brain Networks** (Only the view angle is different from Figure 3 in the Manuscript). **A** – Representation of the centroids obtained for each k-means clustering solution with K ranging between 2 and 20. Centroids are represented in cortical space, rendering only the ROIs whose BOLD phase is shifted $> \pm \pi/2$ with respect to the leading direction. ROIs are coloured according to the reference Functional Brain Network (shown in B) to which they most significantly relate with. Pearson's r and corresponding p-value are reported as a title. A conservative threshold of p<0.05/K was set to correct for FWER. Centroids not significantly overlapping with any of the reference functional networks are coloured in black. **B** - Reference functional brain networks (Yeo et al., 2011).

Supplementary Material

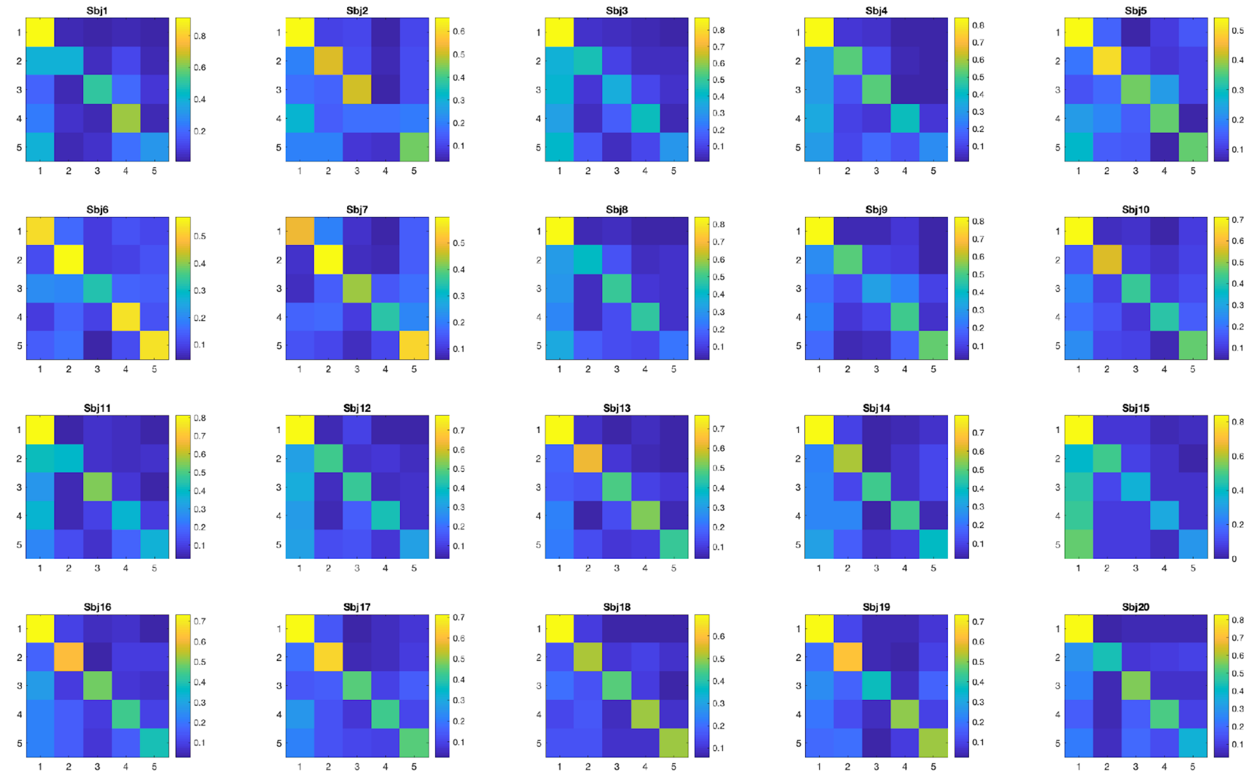

SI Figure 2 – Example of individual Transition Matrices from a representative set of fMRI experiments (participants 1 to 20, LR session) for clustering solution K =5. The transition matrix is an asymmetric matrix where off-diagonal entries represent transition from a given state (lines) to another state (columns) and diagonal entries describing self-transitions.

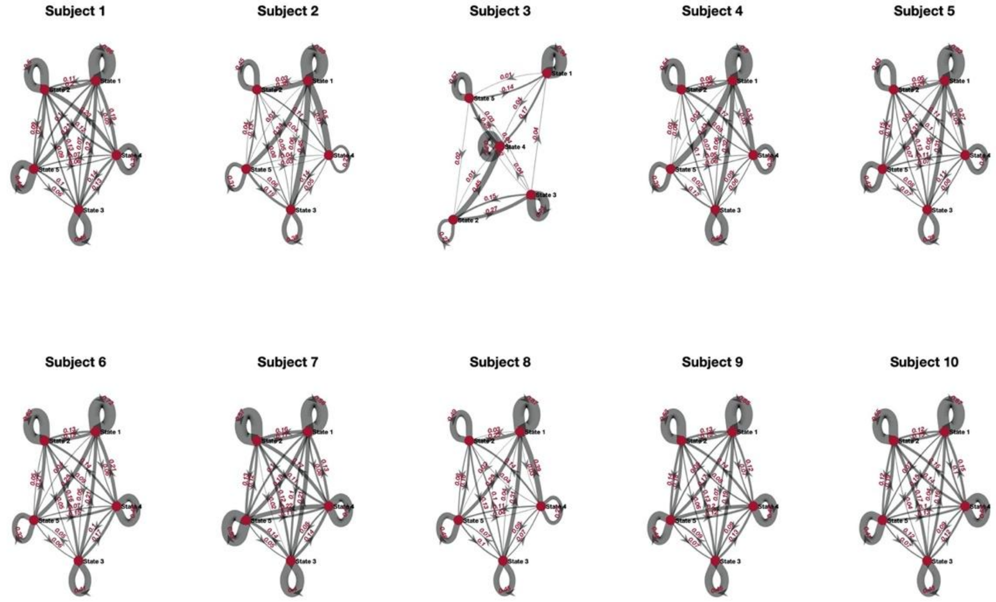

SI Figure 3 – Representation of each fMRI experiment (participants 1 to 10) as a Transition Graph for clustering solution K =5. Edges are weighted by the probability of transition.

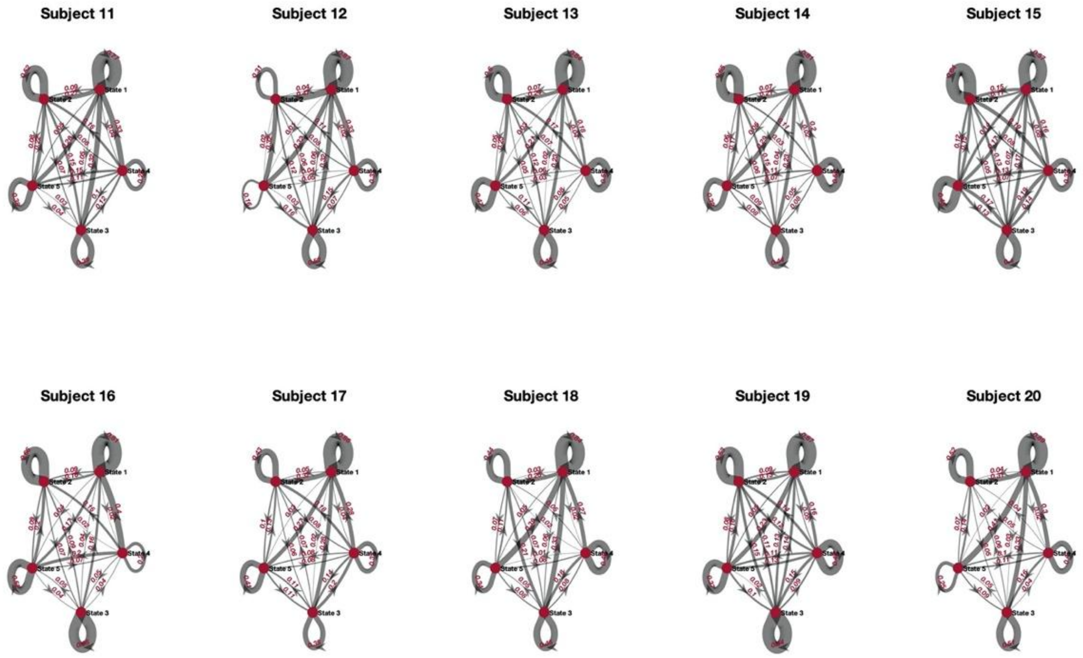

SI Figure 4 – Representation of each fMRI experiment (participants 11 to 20) as a Transition Graph for clustering solution K =5. Edges are weighted by the probability of transition.

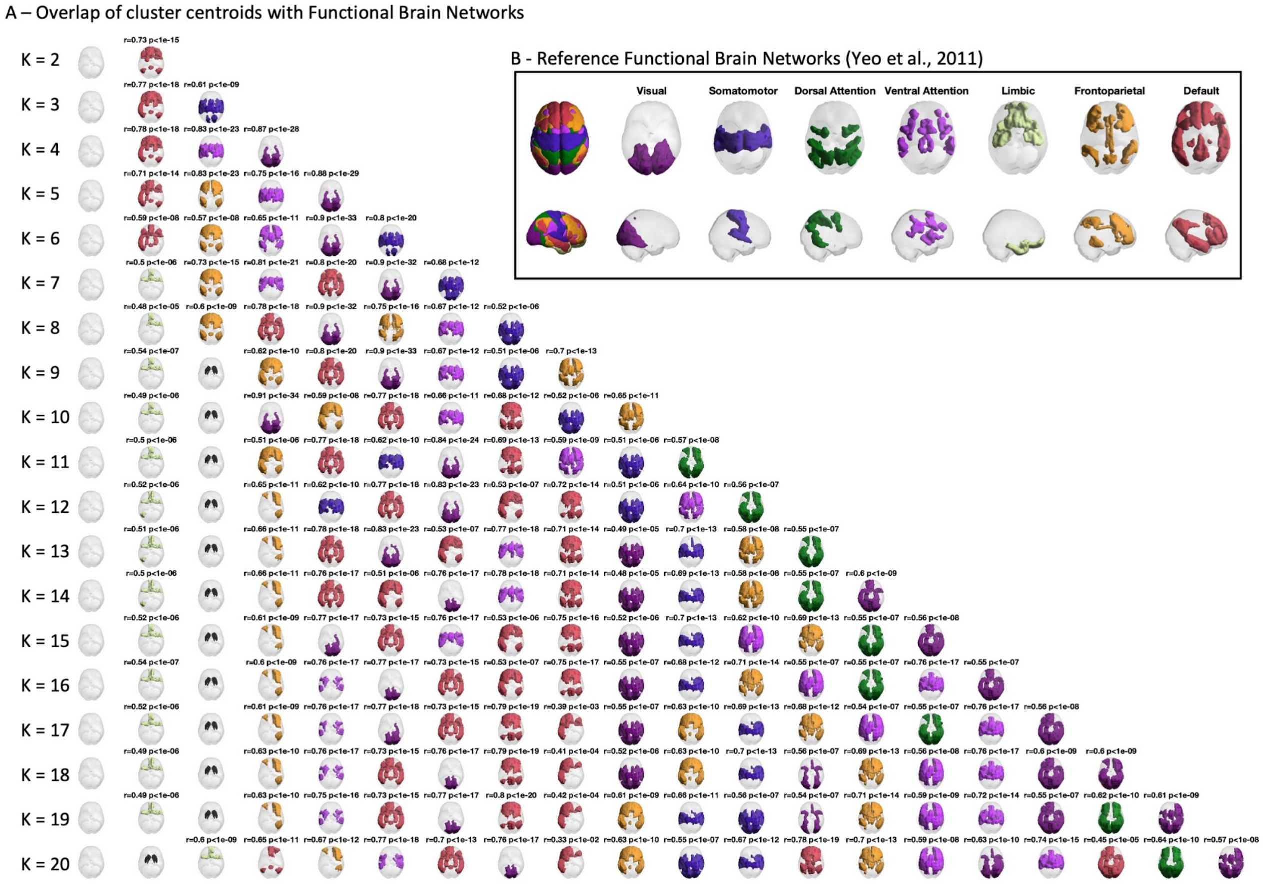

SI Figure 5– **Overlap of Cluster Centroids (Top-view) with reference Functional Brain Networks obtained** for *filtered* BOLD timeseries [0.04-0.07Hz]. A – Representation of the centroids obtained for each k-means clustering solution with K ranging between 2 and 20. Centroids are represented in cortical space, rendering only the ROIs whose BOLD phase is shifted $> \pm \pi/2$ with respect to the leading direction. ROIs are coloured according to the reference Functional Brain Network (shown in B) to which they most significantly relate with. Pearson's r and corresponding p-value are reported as a title. A conservative threshold of p<0.05/K was set to correct for FWER. Centroids not significantly overlapping with any of the reference functional networks are coloured in black. B Reference functional brain networks (Yeo et al., 2011).

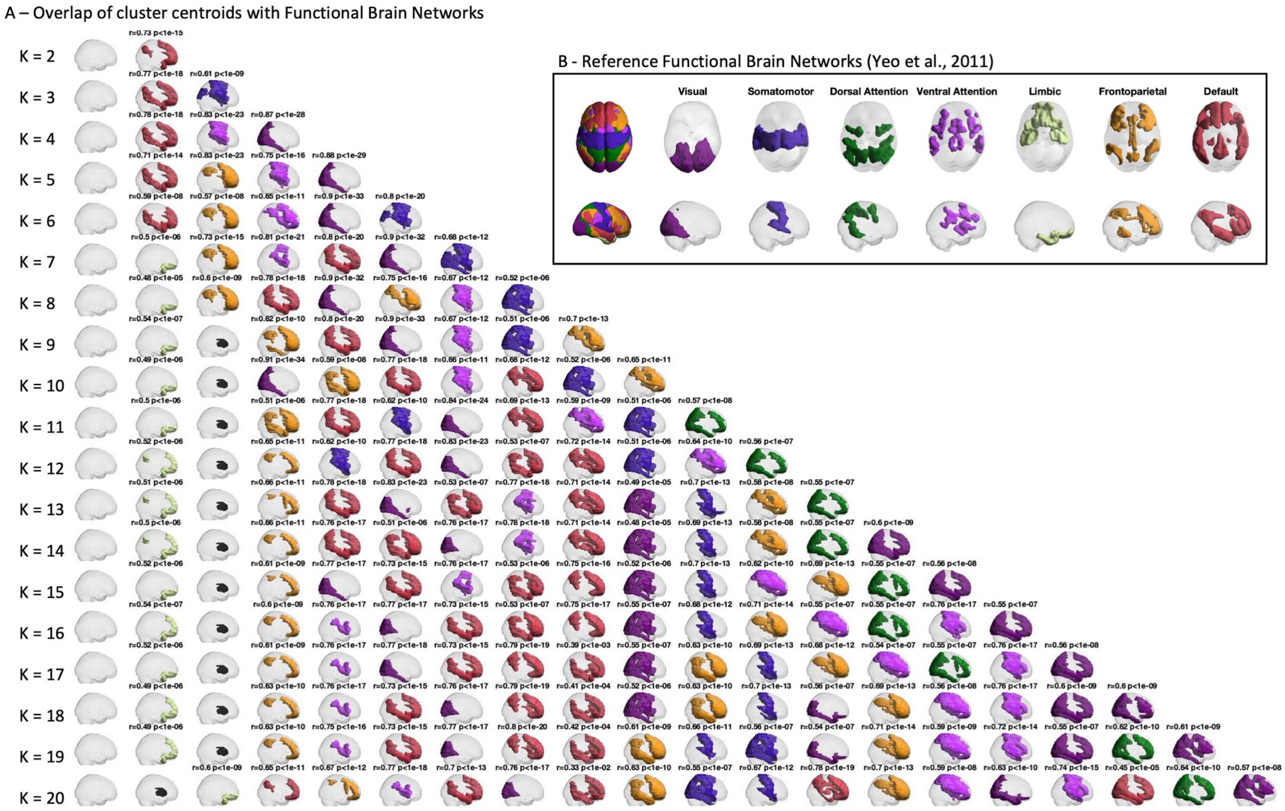

SI Figure 6– **Overlap of Cluster Centroids (Side-view) with reference Functional Brain Networks obtained** for *filtered* BOLD time series [0.04-0.07Hz]. A – Representation of the centroids obtained for each k-means clustering solution with K ranging between 2 and 20. Centroids are represented in cortical space, rendering only the ROIs whose BOLD phase is shifted $> \pm\pi/2$ with respect to the leading direction. ROIs are coloured according to the reference Functional Brain Network (shown in B) to which they most significantly relate with. Pearson's r and corresponding p-value are reported as a title. A conservative threshold of $p<0.05/K$ was set to correct for FWER. Centroids not significantly overlapping with any of the reference functional networks are coloured in black. B Reference functional brain networks (Yeo et al., 2011).

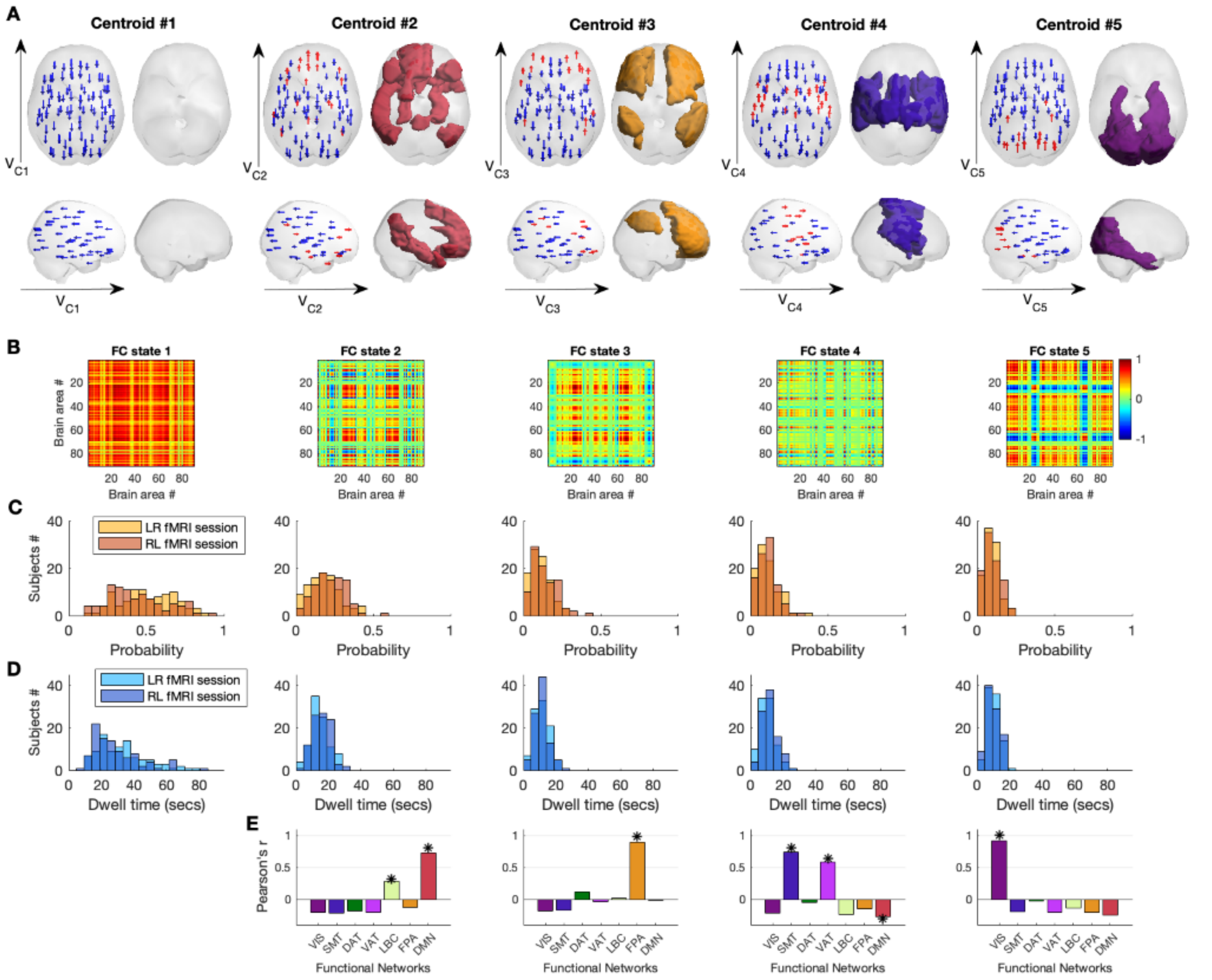

SI Figure 7 – Repertoire of BOLD Phase Locking States obtained using K = 5 for band-pass filtered BOLD timeseries (0.04-0.07Hz). A – BOLD Phase Locking states represent recurrent patterns of BOLD phase alignment across the whole brain. Each centroid is a vector V_C of size 1xN whose elements indicate how each brain area projects into it. Each centroid is represented in the brain in two different ways: (left) by placing an arrow at the centre of gravity of each brain area and setting its size, direction and colour according to the magnitude and sign of the corresponding element in V_C (colouring in red for positive projections into V_C, and blue otherwise). (right) Rendering all brain areas with positive values in V_C coloured according to the functional network to which they show maximal overlap (see panel E below). B Phase-locking states computed as an outer product of the centroid vector V_C and its transpose. C, Histogram of probability of occurrence of each states for all the subjects. The colours represent the different scanning session (LR and RL). D, Histogram of dwell time of each states for all the subjects. The colours represent the different scanning session (LR and RL). E - Correlation between each BOLD PL state and the seven networks of intrinsic functional connectivity from (Yeo et al., 2011).

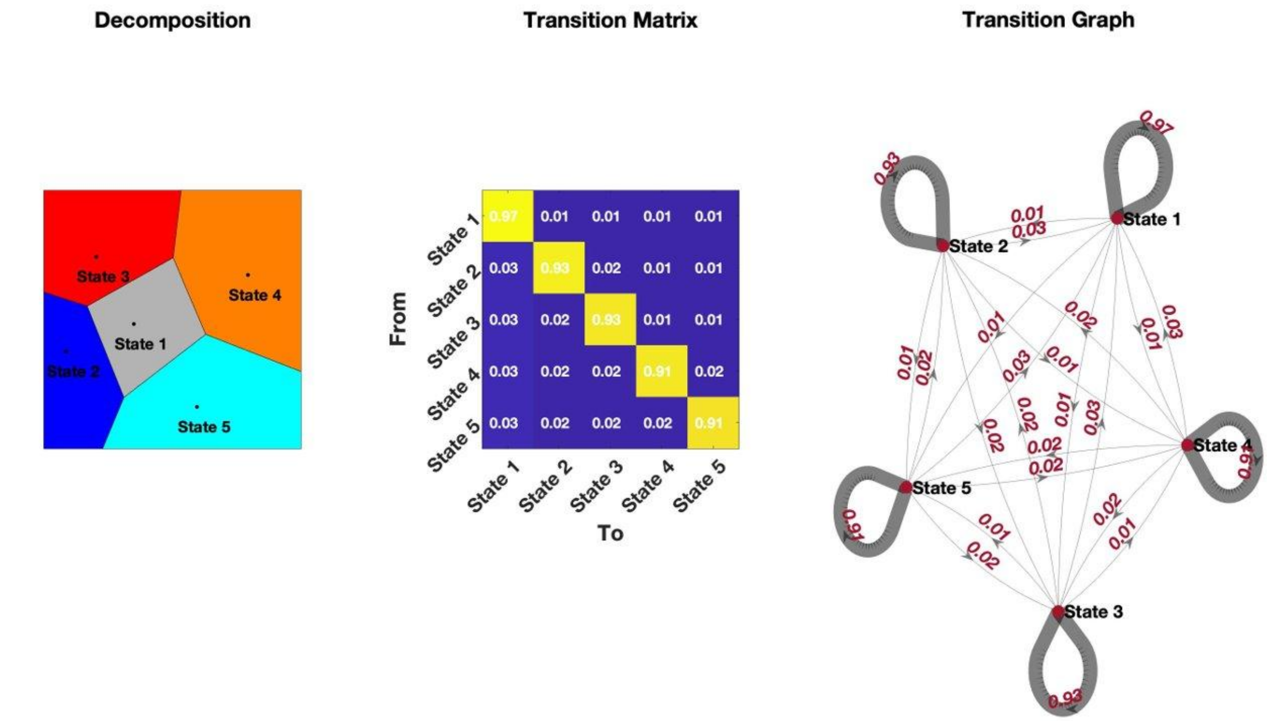

SI Figure 8 – Trajectory of brain activity in state-space (k=5) for filtered timeseries (0.04-0.07Hz). **Left**, Phase space of observations (N-dimensional space where N is the number of brain regions) is decomposed (using K-means clustering) into a meaningful number of subsets which are characterized by their centroids. **Middle**, Transition matrix quantifying the probability of the trajectory transiting from one state to another as defined in Equation 4. **Right**, Transition Graph is constructed from transition matrix W where edges $\alpha \rightarrow \beta$ are directed and weighted with weight $W_{\alpha\beta}$.

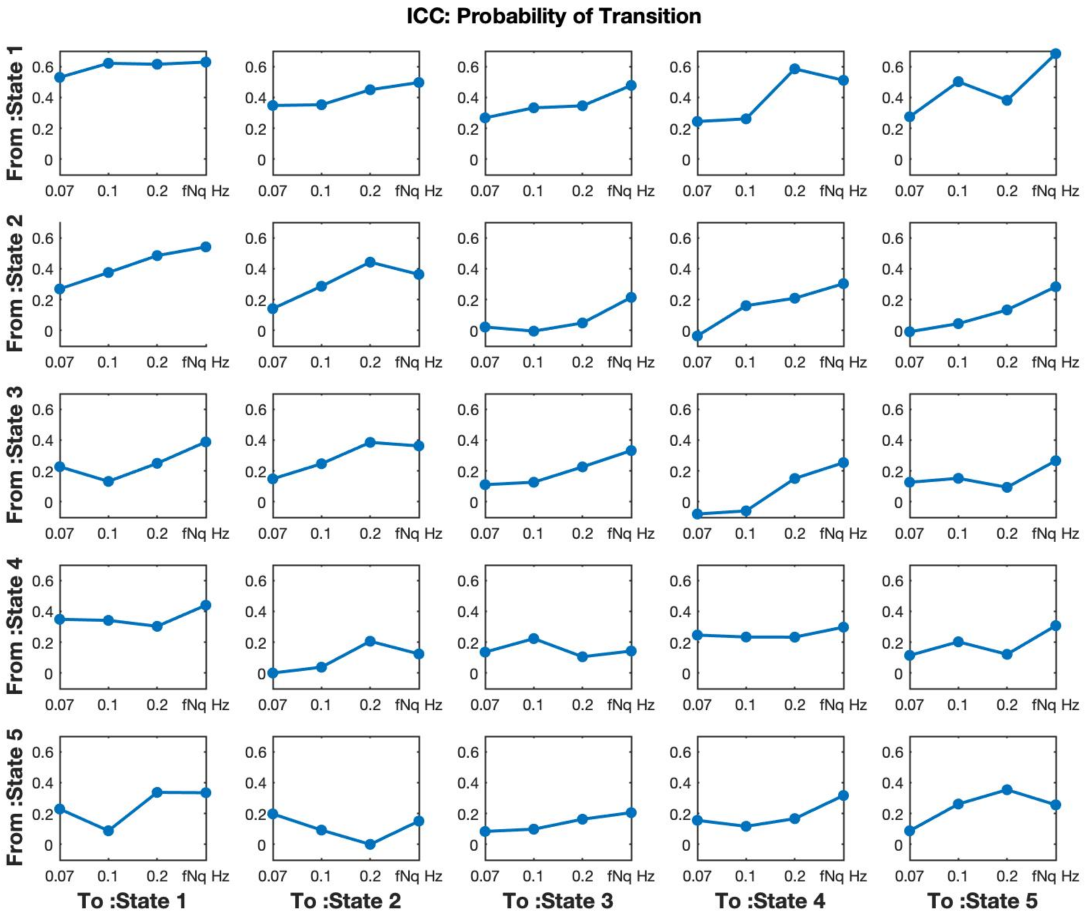

SI Figure 9: Effect of temporal filtering on the ICC of Transition Probabilities. As can be seen the inclusion of higher frequencies improves the ICC values in most cases.

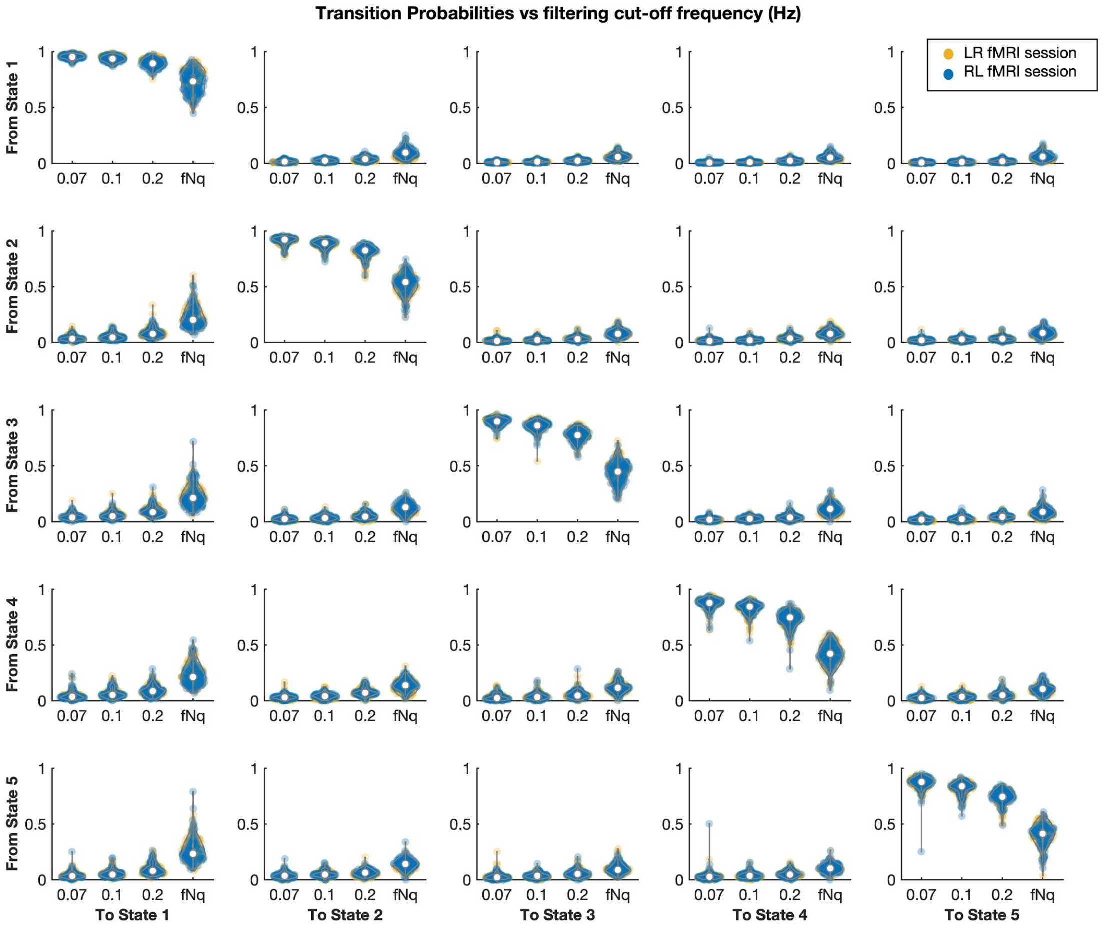

SI Figure 10: Transition Probabilities across participants and for the two sessions (LR and RL). As can be seen, the inclusion of the faster frequency components decreases the probabilities of remaining in the same state (also decreasing the Dwell Times) and increases the probabilities of transition between states, consistently in the 2 sessions.

References:

Yeo, B. T. T., Krienen, F. M., Sepulcre, J., Sabuncu, M. R., Lashkari, D., Hollinshead, M., … Buckner, R. L. (2011). The organization of the human cerebral cortex estimated by intrinsic functional connectivity. *Journal of Neurophysiology*, *106*(3), 1125–1165. https://doi.org/10.1152/jn.00338.2011
